# Health system utilization and perceived quality among adults in Lao PDR: evidence from a nationally representative phone survey

**DOI:** 10.1186/s12889-024-18039-2

**Published:** 2024-02-22

**Authors:** Amit Aryal, Emma Clarke-Deelder, Souksanh Phommalangsy, Sengchanh Kounnavong, Günther Fink

**Affiliations:** 1 Swiss Tropical and Public Health Institute, Allschwil, Switzerland; 2 University of Basel, Basel, Switzerland; 3 Lao Tropical and Public Health Institute, Vientiane, Lao PDR

**Keywords:** Health care systems, Quality of care, Health care utilization

## Abstract

**Background:**

The World Health Organization (WHO) defines quality health services as being effective, safe, people-centered, timely, equitable, integrated and efficient. It is critical to understand people’s perspectives and care experiences to measure progress against these goals. However, many low- and middle-income governments do not routinely collect such information. In this study, we aim to measure health systems performance from the perspective of the adult population of users and non-users in Lao PDR.

**Methods:**

Using the People’s Voice Survey (PVS), a novel phone-based survey designed to integrate people’s voices into primary care performance measurement, we conducted a cross-sectional survey of the general adult (18+) population in Lao PDR in 2022. We analyzed health care utilization patterns, user-reported quality of care, and coverage of key preventive health services. Data from the most recent MICS survey was used to create sampling weights generating nationally representative estimates.

**Results:**

A total of 2007 adults completed interviews in approximately 3.5 months. About two thirds (65%) of respondents reported visiting a health facility in the past year and, of these, the majority (61%) visited a hospital as opposed to a health center or clinic. Among those that recently visited health facilities, 28% rated their experience as “poor” or “fair”. 16% had unmet need for care and 12% reported discrimination during treatment in the past year. 12% of women over 50 years old reported receiving a mammogram and 59% of adults reported receiving blood pressure screening in the previous year.

**Conclusions:**

The study presents data from the first nationally representative survey in Lao PDR to measure health system performance. The results indicate that, despite progress towards universal coverage of health insurance in Lao PDR, significant gaps remain, particularly with respect to bypassing of primary care facilities, significant unmet need for care, experiences of discrimination, and overall low perceptions of quality of care.

**Supplementary Information:**

The online version contains supplementary material available at 10.1186/s12889-024-18039-2.

## Background

Despite increased coverage of essential health care services in low-and middle-income countries (LMICs) as part of their Sustainable Development Goal (SDG) commitments, 5 million people die each year from treatable conditions due to poor quality health systems and an additional 3.6 million lose their lives because of poor access to health care [1]. There is evidence that both technical quality of care (i.e., care that is in line with evidence-based clinical guidelines) and non-technical quality of care (i.e., care that is respectful of patients) play an important role in shaping health care utilization and, ultimately, health outcomes [2, 3].

To improve health system performance, it is important to understand how the population uses health care services and how satisfied the population is with the quality of the services available. However, data on general health service utilization patterns and satisfaction across the population is scarce in Lao PDR as in other low-resource settings [4, 5]. Research that is available is often limited to vertical or siloed health programs (e.g., maternal health), making it difficult to draw generalizations across the health system and the larger population’s health needs [4, 6, 7]. Furthermore, many studies measure patient perspectives using exit surveys, which, by design, exclude members of the population who forego care [8–10]. Last, satisfaction can be difficult to measure and interpret with some studies showing unexpectedly high rates of satisfaction even when the quality of health services is objectively poor [11–13].

Like many countries, Lao PDR has made a commitment to achieve Universal Health Coverage (UHC), defined as universal access to affordable and high quality health care services for all [14]. It has rolled out a national health insurance scheme that has reached near universal coverage [15]. The achievement of UHC, however, will require improvements in health system performance that address not only gaps in access to care but also gaps in quality of care.

In this study, we aimed to measure health system performance in Lao PDR from the perspective of the general adult population and draw attention to the gaps in health care quality. We used the People’s Voice Survey (PVS), a newly developed tool that enables rapid assessment of health system performance through a population-based mobile phone survey. It focuses on health system use patterns, care experience, health system competence, trust and confidence in the health system, and people’s perceptions of quality of both health care quality and the larger health system [16]. By randomly sampling from the adult population, rather than measuring quality at the point of care, the PVS captures the perspectives of both health system users and non-users [16]. The PVS was developed through a multi-country collaborative process involving researchers, policymakers, and health system stakeholders, as described in previous work [16].

## Methods

### Setting

Lao PDR is a lower-middle income country in southeast Asia with a population of approximately 7 million and a per capita GDP income of USD 2536 [17]. The health care system in Lao includes publicly-managed health centers, publicly-managed hospitals, and a growing number of private clinics and hospitals [5]. Health centers are responsible for providing primary care through on-site and outreach to remote communities and are supervised by district health offices [5].

Childbirth care and health services for children under the age of five are free of charge. In addition, a national health insurance scheme has been in operation since 2017, providing coverage to the general population [18]. Civil servants, police, and army personnel are eligible for additional social security measures, and private health insurance options are available through employment-based schemes [18].

Lao PDR has committed to achieving Universal Health Care (UHC) by 2025 and has adopted a “5-Goods 1-Satisfaction” policy to improve quality of care, which focuses on better diagnosis and treatment to improve patient outcomes [19]. Despite these efforts, Lao PDR faces high rates of non-communicable diseases, infectious diseases such as malaria, dengue, and tuberculosis, as well as regionally high rates of maternal and newborn mortality [20, 21].

### Study design

We conducted a phone-based cross-sectional survey among the general adult population aged 18 and higher in Lao PDR. Age-, gender-, residence- and region-specific sampling targets were used to ensure nationally representative data. We measured health care utilization patterns, perceptions of quality of care during the most recent visit to a health facility, self-reported experience of discrimination and medical errors in the previous year, and coverage preventive health services.

### Participants

Respondents were screened for age and language prior to starting the interview. People less than 18 or not able to communicate in any of the three main languages (Lao, Hmong and Khmu) were excluded from the survey. All respondents were informed about the survey and provided informed consent verbally before they started answering questions.

### Data collection

Data collection for the survey took place between 9 May 2022 and 19 August 2022. The survey was translated to Lao, the official language of Lao PDR, and additionally to Khmu and Hmong, the two most commonly spoken ethnic minority languages. Five enumerators were trained on the Lao version of the survey instrument over a 5-day period. One of enumerators was fluent in writing and speaking in Hmong and another in Khmu. Both ethnic minority language speaking enumerators translated the questionnaire into their respective ethnic language, which were validated through verbal back-translations. Prior to the survey, cognitive interviews were conducted with 5 people in each of the three languages, using a combination of the “think aloud” and the “scripted probing” techniques [22]. Cognitive interviews were conducted in both urban and rural areas to ensure that survey questions worked well with a range of participants. The interviews helped identify appropriate terminology for concepts such as “health services”, “health care providers,” and the larger “health system,” while ensuring that the original intent of the questions was understood by local populations. A pilot study was conducted to ensure feasibility of study procedures and the questionnaire. Following the pilot of 46 completed interviews, we clarified the recruitment script and made minor changes to the survey coding to improve flow. We also used the pilot to evaluate the extent to which participants remained focused throughout the survey, by asking a set of repeated questions at the end of the survey. We found that responses at the beginning and end of the survey were consistent, suggesting that respondents remained focused (as opposed to entering random numbers as they reached the latter questions).

While phone-based surveys present an attractive means for data collection, there are some potential errors that can result in biased sample estimates, which need to be minimized [23]. These errors primarily arise because of non-coverage (i.e. differences between those who own and those who do not own mobile phones) and non-response error (differences between those who respond and those who do not respond to surveys) [24]. More than 90% of households in Lao PDR have a phone, which makes it an attractive country to deploy phone-based survey methods and minimize non-coverage error [21, 23]. Strategies recommended by Nagpal et al. to minimize additional sources of non-coverage and non-response error were incorporated in the design, implementation and analysis phases of the study [24]. Specifically, to ensure that the sample was representative of the population, a target sample size was defined for group stratified by age (18 to 45, 45 and higher), gender (men, women), geographic area (urban, rural) and region (north, central, south) based on a prior nationally-representative household survey [21]. We regularly monitored the sample size in each strata relative to the target and sought to reach at least 80% of the target sample for each demographic group. We developed a structured callback protocol to improve response rates and minimize non-response errors, where each sampled number was called at least five times at different times of the day. Additional file 1 shows breakdown of call outcomes for phone numbers called during the survey.

Our target sample was 2000 completed interviews, consistent with national phone surveys such as Afrobarometer and Latinobarometer [25, 26]. Mobile phone numbers used for the study were purchased from Sample Solutions B. V., an international market research company.

### Measurement

Study outcomes included type of health facility used for the most recent visit in the previous year, user experience and quality rating during the most recent visit to a health facility, and coverage of preventive health services. To measure the use of health facilities in the previous year, we asked the following question to those that reported going to a health facility in previous 12 months: *Did you go to a hospital, health center or clinic for your last visit?* We asked a follow-up to those that went to a hospital to ascertain if it was public or privately managed. Use of health services through other means (telemedicine, home visits, overnight stays) in the previous year were measured based on responses to similarly phrased questions: *How many times did you use the phone or computer to seek counseling or care with a health care provider in the past 12 months?* Unmet need for health care was measured based on responses to the following question: *In the past 12 months, was there a time when you had a health problem and needed medical attention, but you did not get healthcare from a provider?* Care experiences in the previous year were measured by asking a closed question with Yes or No responses: *In the last 12 months, were you treated unfairly or discriminated against by a doctor, nurse, or another healthcare provider?* Like with others questions, respondents could refuse to respond with Yes or No. To measure the use of preventive health services, we asked people if they had received a range of tests in the previous year: blood glucose, cholesterol, vision check, teeth check, care for depression, anxiety or mental health conditions, mammograms (women over 50) and cervical cancer screening tests (all adult women) in the previous year. The following phrasing was used: *Can you please tell me if you have received any of the following examinations in the past 12 months from any healthcare provider: Had your blood pressure tested?* Responses recorded were “Yes”, “No”, “Do not know” or “Refused”.

We measured user experience during the most visit to health facility (waiting time to see provider, consultation time with health care provider, any experience of medical error, and any experience unfair treatment or discrimination during treatment) and user-reported quality of care during the most recent visit to a health facility (overall and individual domains) Questions to assess perceptions of quality were framed in the following way: *How would you rate the overall quality of care you received?* Ratings for quality of care were based on a 5-point categorical response scale (excellent, very good, good, fair, and poor). We considered “excellent” or “very good” ratings as perceptions of high-quality and “fair” or “poor” ratings as low quality.

### Statistical methods

Post-stratification weights were used to adjust raw data to reflect population demographic characteristics using an iterative proportional fitting algorithm known as raking [27]. Population parameters were calculated using weighted data from the 2017 Multiple Indicator Cluster Survey (MICS) in Lao PDR [21] and scaled to the total population size of 7,427,615 in 2022 [28]. The sample weights were chosen to match the national population with respect to age structure (proportion of adult population 18–29, 30–49, 50+), gender (men or women), region (Central, North, or South), urbanity (urban or rural), urban gender share and urban age structure.

We started our analysis by describing the characteristics of the weighted and unweighted samples, and mapping the geographic distribution of the sample. Next, we explored respondents’ interactions with the health system in the previous year: the proportion that visited a health facility at least once in the previous year; the type of facility visited; the proportion that had at least one telemedicine call, home visit and overnight stay in a hospital in the previous year; and the proportion that had unmet need for health care. We then described respondents’ perceptions of health care quality, including: the proportions that reported experiencing low quality of care (fair or poor quality rating) during the most recent visit to a health facility (overall, and focusing on specific attributes of care); the proportion that reported experiencing long wait times (more than 45 minutes) and short consultation times (less than 15 minutes); the proportion that reported experiencing discrimination in the previous year; and the proportion that reported experiencing a medical error in the previous year. Finally, we described coverage of preventive health services. All analyses are broken down by age and gender.

## Results

A total of 11,835 unique phone numbers were called, yielding 2007 completed interviews. The response rate, 17%, was comparable to nationally representative phone surveys carried out in diverse LMIC countries both in Asia and Africa [29, 30]. Table 1 shows the demographic characteristics of the weighted and unweighted sample compared to population estimates from MICS conducted in 2017. The weighted sample was similar to the population estimates from the MICS: 52% were women; 72% were below the age of 50; 66.3% lived in rural areas; nearly 50% lived in the central region and 56% of the population had primary or less education. The weighted sample, however, had higher proportion of Lao-Tai relative to the MICS estimate (71 to 67%) and Hmong-mien (11 to 8%) and a lower proportion of Mon-Khmer (16 to 22%).

**Table 1:** Sample description

Characteristics	Unweighted sample N (%)	Weighted sample N (%)	Population estimate from Lao PDR MICS 2017 (%)
**Gender**			
Men	1137 (56.5%)	973 (48.5%)	48.5%
Women	870 (43.4%)	1034 (51.5%)	51.5%
**Age group**			
18–29	530 (26.4%)	623 (31.0%)	31.0%
30–39	423 (21.1%)	462 (23.0%)	23.0%
40–49	432 (21.5%)	352 (17.5%)	17.5%
50–59	430 (21.4%)	307 (15.3%)	15.3%
60+	192 (9.6%)	263 (13.1%)	13.1%
**Location**			
Urban	1183 (58.9%)	682 (34.0%)	33.8%
Rural	824 (41.0%)	1324 (66.0%)	66.3%
**Region**			
North	518 (25.8%)	634 (31.6%)	31.6%
Central	1257 (62.6%)	995 (49.6%)	49.6%
South	232 (11.6%)	377 (18.8%)	18.8%
**Educational achievement**			
Primary of less	309 (15.4%)	1131 (56.4%)	56.4%
Lower or upper secondary	871 (43.4%)	582 (29.0%)	29.0%
Post-secondary or tertiary	827 (41.2%)	293 (14.6%)	14.6%
**Ethnicity**			
Lao-Tai	1590 (79.4%)	1420 (71.0%)	66.7%
Mon-Khmer	220 (11.0%)	318 (16.0%)	22.0%
Hmong-Mien	151 7.54%)	223 (11.2%)	7.7%
Chinese-Tibetan	14 (0.70%)	14 (0.68%)	2.6%
Other	28 (1.40%)	27 (1.34%)	1.0%

**Fig. 1:**
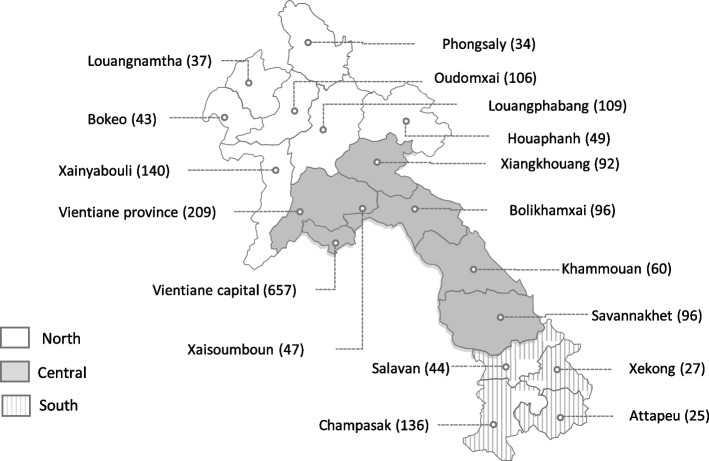
Completed calls by province and region

Figure 1 shows the breakdown of completed calls by province and region. Participants were randomly sampled from each of the provinces with the largest proportion (63%) coming from the central region and 12% from the southern region, similar to the population distribution.

Table 2 shows health system utilization patterns of the adult populations in the previous year. Almost two-thirds (65%) of adults visited a health facility within the previous year and majority of these visits were to government hospitals (61%) and government health centers (17%). A smaller proportion visited private clinics (20%) and private hospitals (2%). People also accessed health care by other means: 4% received care via telemedicine, 7% were visited by health workers and 11% had at least one overnight stay at a hospital. There was a relatively high rate of unmet need for care (16%), particularly for young women between the ages of 18 to 29 (30%), who were more likely to have higher unmet need than men (18%) in the same age category.

**Table 2:** Health system utilization patterns

	Sex	N (survey responses)	Total	By age					*p*-value [1]
				18–29	30–39	40–49	50–59	60+	
**1. At least 1 facility visit in previous year**									
	All	1990	**65%**	63%	68%	66%	58%	70%	0.015
	Women	865	**68%**	69%	69%	69%	65%	65%	0.833
	Men	1125	**62%**	56%	67%	63%	53%	75%	< 0.001
**2. Recent health facility visited**									
Government hospital	All	1307	**61%**	67%	53%	64%	62%	57%	0.002
	Women	588	**64%**	71%	59%	65%	75%	43%	< 0.001
	Men	719	**57%**	61%	46%	62%	49%	69%	< 0.001
Government health centre	All	1307	**17%**	15%	25%	10%	18%	14%	< 0.001
	Women	588	**15%**	12%	22%	11%	12%	19%	0.036
	Men	719	**19%**	20%	28%	10%	24%	9%	< 0.001
Private hospital	All	1307	**2%**	1%	3%	3%	1%	2%	0.288
	Women	588	**2%**	2%	1%	4%	1%	3%	0.504
	Men	719	**2%**	0%	5%	2%	1%	2%	0.038
Private clinic	All	1307	**20%**	16%	19%	21%	19%	27%	0.059
	Women	588	**18%**	15%	18%	17%	12%	36%	0.001
	Men	719	**21%**	18%	20%	25%	26%	18%	0.350
Other	All	1307	**1%**	1%	0%	2%	0%	1%	0.171
	Women	588	**1%**	0%	0%	3%	0%	0%	0.005
	Men	719	**1%**	1%	0%	0%	0%	1%	0.608
**3. At least 1 telemedicine call**									
	All	1989	**4%**	3%	3%	8%	5%	4%	0.004
	Women	864	**5%**	2%	3%	12%	7%	7%	< 0.001
	Men	1125	**3%**	5%	3%	4%	3%	1%	0.252
**4. At least 1 home visit by health worker**									
	All	1996	**7%**	7%	5%	8%	8%	10%	0.188
	Women	866	**7%**	9%	5%	11%	4%	6%	0.092
	Men	1130	**7%**	4%	5%	4%	12%	14%	< 0.001
**5. At least 1 overnight stay in hospital**									
	All	1988	**11%**	12%	12%	9%	7%	13%	0.050
	Women	865	**13%**	17%	15%	9%	9%	11%	0.069
	Men	1123	**9%**	7%	8%	9%	5%	16%	0.008
**6. Did not use care when needed (unmet need)**									
	All	2004	**16%**	24%	11%	19%	10%	11%	< 0.001
	Women	869	**20%**	30%	12%	25%	9%	12%	< 0.001
	Men	1135	**13%**	18%	10%	13%	11%	9%	0.023

[1] ANOVA test for equality of proportions across age groups.

About one quarter (28%) of adults gave an overall low rating to the quality of care that they received in their most recent visit to a health facility (Table 3). Young adults [18–29] were more likely to give low ratings across all aspects of quality compared to older adults. They were particularly dissatisfied with waiting time (38%) and the quality of equipment and supplies (40%). Nearly two-thirds (63%) of consultations with a provider lasted less than 15 minutes and approximately 14% of respondents waited longer than 45 minutes to be seen by a provider and women. 12% reported experiencing discrimination or unfair treatment. As shown in Additional file 2, women experienced discrimination twice as often as men (15% vs 7%). Finally, 5% of respondents reported experiencing medical error in the past year.

**Table 3:** Perceptions of quality of care

	N (survey responses)	Total	By age					
			18–29	30–39	40–49	50–59	60+	*p*-value [1]
**Low quality rating (poor or fair) of care during most recent visit**								
1. Poor or fair rating of overall quality	1326	**28%**	32%	31%	23%	19%	29%	0.004
2. Poor or fair rating of provider skill and knowledge	1322	**23%**	29%	17%	24%	20%	23%	0.011
3. Poor or fair rating of respect shown by provider	1327	**23%**	28%	23%	20%	17%	20%	0.018
4. Poor or fair rating of provider knowledge about previous visits or tests	1292	**21%**	29%	23%	16%	18%	11%	< 0.001
5. Poor or fair rating of provider explanations	1326	**20%**	29%	21%	11%	18%	15%	< 0.001
6. Poor or fair rating of involvement in decisions by provider	1324	**18%**	27%	20%	11%	12%	12%	< 0.001
7. Poor or fair rating of time spent in consultation by provider	1326	**20%**	30%	25%	12%	16%	8%	< 0.001
8. Poor or fair rating of waiting time for provider	1327	**24%**	38%	25%	16%	15%	8%	< 0.001
9. Poor or fair rating of helpfulness of support staff	1318	**21%**	32%	23%	16%	15%	9%	< 0.001
10. Poor or fair rating of equipment and supplies	1317	**35%**	40%	38%	30%	31%	26%	0.003
11. Wait-time to see provider exceeded 45 minutes	1297	**14%**	13%	14%	15%	17%	9%	0.235
12. Consultation time with provider was less than 15 minutes	1293	**63%**	58%	58%	69%	70%	64%	0.007
**Care experience over the past year**								
1. Experienced medical error during treatment	1403	**5%**	8%	5%	3%	3%	2%	0.003
2. Experienced discrimination or unfair treatment by a health worker	1404	**12%**	19%	8%	13%	8%	5%	< 0.001

[1] ANOVA test for equality of proportions across age groups.

**Fig. 2:**
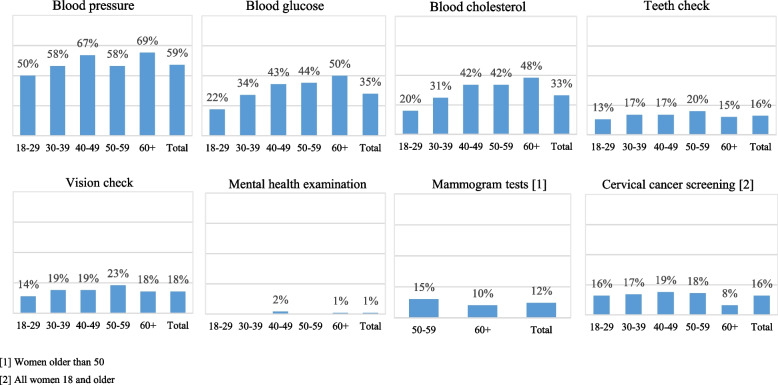
Coverage of preventive health services by age

The coverage of preventive health services varied widely across services and across age groups (Fig. 2). 12% of women above 50 years old were screened for breast cancer by mammogram and 16% of adult women were screened for cervical cancer. Less than 1 in 5 adults received a vision (18%) or teeth (16%) examination in the previous year. Approximately 3 out of 5 adults (59%) received a blood pressure examination, 1 out of 3 (35%) received a blood glucose examination and 1 out of 3 (33%) received a blood cholesterol examination in the past year; higher proportions of both older men and women (see Additional file 3) received the three examinations relative to their younger counterparts. Only 1% of adults received mental health screenings in the past year.

## Discussion

In this study, we present data from the first nationally representative population-focused and mobile-based survey in Lao PDR to measure health system performance. Our results confirm that Lao PDR has made good progress towards universal health coverage [15], but also highlight significant bypassing of health centers, primary care facilities, high unmet need among young (18–29 year olds) women and low perceptions of quality of care, particularly among young adults between 18 and 29 years old.

There are several key results worth highlighting. Consistent with many other studies in LMICs, there are high rates of bypassing of primary care facilities [31, 32]. Despite the fact that there are over 1000 health centers in communities across the country, we found that the population largely relies on hospitals (61%) for their health care needs with another 20% using private clinics and only 17% relying on health centers for care. While there is limited research on bypassing in Lao PDR, preference for hospitals over lower levels of care is likely due to more qualified health personnel, availability of a more comprehensive set of health services, better equipment, and consultation fees that are similar to health centers [33]. Past studies have found that the rate of bypassing is lower when primary health care centers are staffed with competent clinicians, suggesting that patients’ bypassing decisions are – at least to some extent – informed by the quality of care available in primary health centers [32]. Bypassing of primary care facilities in Lao PDR is similar to other countries that face significant quality gaps [32, 34, 35]. Recently published study based on PVS from 14 high, middle and low income countries showed that the use of secondary or higher facilities as usual source of care was among the highest in Lao PDR [36]. Furthermore, similar to Zambia, where a recent study identified high rates of bypassing of primary care [37], Lao PDR has weak gate-keeping policies and allows people to directly access specialists in hospitals [5].

We found high rates of dissatisfaction in quality of care with 1 in 4 (28%) adults rating overall quality of the recently visited facility as poor or fair. When asked to rate specific aspects of their care, participants give the lowest ratings to equipment and supplies (with 35% rating this aspect of care as poor or fair), followed by waiting times (24%), provider knowledge (23%), and provider respect (23%). These ratings are substantially lower than quality ratings seen in similar studies in other LMICs [4, 38] High rates of dissatisfaction with quality of care coupled with relatively high unmet need (16%) may dampen Lao PDR’s near-universal achievement of health insurance coverage. Another concerning finding from the study is the relatively high proportion (12%) of adults reporting discrimination during treatment with even higher rates among young women (24%) between the ages of 18 and 29 (see Additional file 2). While we did not find differences in rates of discrimination among the three main ethnicities (Lao-tai, Mon-khmer and Hmong-mien), women belonging Mon-Khmer ethnic minority group reported higher rates of unmet need for care compared to Lao-tai women (see Additional file 4). Further qualitative exploration to understand underlying reasons for low perception of quality, unmet need for care, discrimination experienced by women, particularly among ethnic minorities, is warranted.

Despite the fact that most adults have accessed health care within the past year, we observed relatively low coverage of critical preventive services. A majority of women are currently not being screened for breast and cervical cancers, the leading causes of morbidity and mortality among women in the country [39]. According to the data presented here, only 12% women older than 50 received mammography and 16% were tested for cervical cancer in the previous year, likely via Pap smear or VIA [40]. Achieving biennial mammography tests would require 50% annual coverage among women older than 50, a 4 times increase from current coverage. To achieve cervical cancer screening once every 3 years, approximately twice the current annual coverage (16%) will be needed. Increased screening for hypertension and diabetes, particularly among adults aged 40 and above also seems desirable.

Our study has several limitations. Firstly, while we adopted strategies by Nagpal et al. [24] to minimize non-coverage and non-response errors, our sample covers only adults with mobile phone access. In theory, sample weighting should address selective responsiveness, but it is possible that some groups are underrepresented as we cannot adjust for unobserved characteristics that might affect response. Secondly, individuals’ responses to the PVS may be affected by biases such as social desirability bias, and may therefore not fully reflect their true perspectives on health system performance. However, even with the possibility of this form of bias, we find that respondents are highly critical of many aspects of health system performance in Laos. Finally, it is important to note that our survey was conducted during the COVID-19 pandemic; the observed utilization patterns, expectations for quality care and confidence in the overall health system are all likely to have been impacted by the pandemic. However, this survey can provide a baseline for future work assessing changes over time as the country recovers from the pandemic.

Overall, our results suggest that additional investments are needed to improve access to high quality health care in Lao PDR. Policy-makers may be influenced to expand hospital capacity in response to their high demand but hospitals already accounts for nearly half of the total annual health expenditures [41]. Expanding the scope and quality of primary care services offered at the health center level may be a more effective and efficient means to deliver high-quality and equitable care while reducing the high burden of out-of-pocket expenditures, which is currently 45% [41]. Policymakers and researchers have called for people-centered health services that are oriented around the needs and aspirations of people and communities; this survey suggests that there is still significant work to be done to meet these ambitious goals in Lao PDR [42].

## Conclusions

The results presented in this study indicate that, despite progress towards universal coverage of health insurance in Lao PDR, significant gaps remain, particularly with respect to bypassing of primary care facilities, significant unmet need for care, experiences of discrimination particularly faced by women, and overall low perceptions of quality of care. In response to these findings, a significant policy effort to upgrade the capacities of health centers to deliver preventive care that is accessible and closer to people’s homes. This may help building trust in the local health systems and improve their perception of quality care of government primary care systems.

### Supplementary Information

**Additional file 1.**

**Additional file 2.**

**Additional file 3.**

**Additional file 4.**

## Data Availability

The datasets used during the current study are available from the Quality Evidence for Health System Transformation (QuEST) on reasonable request.

## Abbreviations

GDP: Gross Domestic Product
Lao PDR: Lao People’s Democratic Republic
Lao TPHI: Lao Tropical and Public Health Institute
LMICs: Low-and middle-income countries
VIA: Visual Inspection with Acetic Acid
MICS: Multiple Indicator Cluster Survey
PVS: People’s Voice Survey
SDG: Sustainable Development Goals
Swiss TPH: Swiss Tropical and Public Health Institute
UHC: Universal Health Coverage
USD: United States Dollar
WHO: World Health Organization
