# Supplementary figures and images for: Health system utilization and perceived quality among adults in Lao PDR: evidence from a nationally representative phone survey
Source: BMC Public Health. 2024 Feb 22;24:565. doi: 10.1186/s12889-024-18039-2 (PMC10882776; doi:10.1186/s12889-024-18039-2)

Additional file 1. Sample flowchart


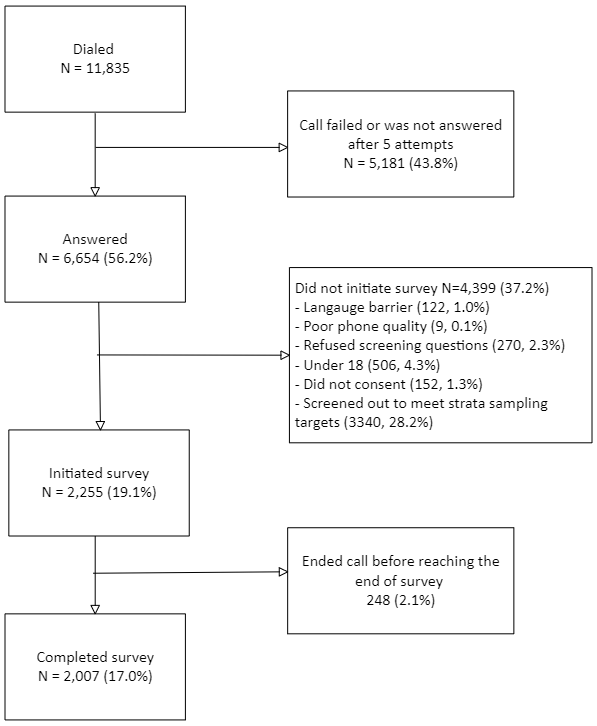

Supplement: Supplementary file 1 — Additional file 1. [file 12889_2024_18039_MOESM1_ESM.docx]
