# Supplementary material for: Health system utilization and perceived quality among adults in Lao PDR: evidence from a nationally representative phone survey
Source: BMC Public Health. 2024 Feb 22;24:565. doi: 10.1186/s12889-024-18039-2 (PMC10882776; doi:10.1186/s12889-024-18039-2)
Supplement: Supplementary file 2 — Additional file 2. [file 12889_2024_18039_MOESM2_ESM.docx]

Additional file 2. Perceptions of quality of care by age and sex

|  | **Sex** | **N**  **(survey responses)** | **Total** | **By age** | | | | | **p-value^[1]^** |
| --- | --- | --- | --- | --- | --- | --- | --- | --- | --- |
|  |  |  |  | **18-29** | **30-39** | **40-49** | **50-59** | **60+** |  |
| **Low quality rating (poor or fair) of care during most recent visit** | | | | | | | | | |
| 1. Poor or fair rating of overall quality | All | 1326 | 28% | 32% | 31% | 23% | 19% | 29% | 0.004 |
|  | Women | 604 | 26% | 31% | 29% | 28% | 21% | 14% | 0.053 |
|  | Men | 722 | 30% | 34% | 34% | 18% | 16% | 42% | <0.001 |
|  |  |  |  |  |  |  |  |  |  |
| 2. Poor or fair rating of provider skill and knowledge | All | 1322 | 23% | 29% | 17% | 24% | 20% | 23% | 0.011 |
|  | Women | 604 | 24% | 29% | 18% | 26% | 15% | 26% | 0.073 |
|  | Men | 723 | 23% | 28% | 17% | 23% | 25% | 21% | 0.142 |
|  |  |  |  |  |  |  |  |  |  |
| 3. Poor or fair rating of respect shown by provider | All | 1327 | 23% | 28% | 23% | 20% | 17% | 20% | 0.018 |
|  | Women | 603 | 21% | 28% | 20% | 22% | 12% | 13% | 0.012 |
|  | Men | 720 | 24% | 28% | 25% | 18% | 22% | 27% | 0.250 |
|  |  |  |  |  |  |  |  |  |  |
| 4. Poor or fair rating of provider knowledge about previous visits or tests | All | 1292 | 21% | 29% | 23% | 16% | 18% | 11% | <0.001 |
|  | Women | 593 | 19% | 28% | 22% | 14% | 8% | 13% | <0.001 |
|  | Men | 699 | 23% | 31% | 23% | 18% | 28% | 10% | <0.001 |
|  |  |  |  |  |  |  |  |  |  |
| 5. Poor or fair rating of provider explanations | All | 1326 | 20% | 29% | 21% | 11% | 18% | 15% | <0.001 |
|  | Women | 604 | 23% | 33% | 24% | 12% | 19% | 13% | <0.001 |
|  | Men | 722 | 18% | 24% | 17% | 11% | 17% | 17% | 0.075 |
|  |  |  |  |  |  |  |  |  |  |
| 6. Poor or fair rating of involvement in decisions by provider | All | 1324 | 18% | 27% | 20% | 11% | 12% | 12% | <0.001 |
|  | Women | 603 | 19% | 28% | 20% | 12% | 7% | 14% | <0.001 |
|  | Men | 721 | 18% | 26% | 21% | 10% | 18% | 9% | <0.001 |
|  |  |  |  |  |  |  |  |  |  |
| 7. Poor or fair rating of time spent in consultation by provider | All | 1326 | 20% | 30% | 25% | 12% | 16% | 8% | <0.001 |
|  | Women | 604 | 22% | 33% | 27% | 12% | 10% | 9% | <0.001 |
|  | Men | 722 | 19% | 26% | 21% | 12% | 21% | 7% | <0.001 |
|  |  |  |  |  |  |  |  |  |  |
| 8. Poor or fair rating of waiting time for provider | All | 1327 | 24% | 38% | 25% | 16% | 15% | 8% | <0.001 |
|  | Women | 605 | 27% | 43% | 26% | 19% | 16% | 9% | <0.001 |
|  | Men | 722 | 19% | 30% | 24% | 12% | 14% | 8% | <0.001 |
|  |  |  |  |  |  |  |  |  |  |
| 9. Poor or fair rating of helpfulness of support staff | All | 1318 | 21% | 32% | 23% | 16% | 15% | 9% | <0.001 |
|  | Women | 597 | 23% | 36% | 23% | 14% | 17% | 6% | <0.001 |
|  | Men | 721 | 20% | 26% | 24% | 19% | 13% | 12% | 0.008 |
|  |  |  |  |  |  |  |  |  |  |
| 10. Poor or fair rating of equipment and supplies | All | 1317 | 35% | 40% | 38% | 30% | 31% | 26% | 0.003 |
|  | Women | 599 | 32% | 35% | 41% | 24% | 23% | 25% | 0.006 |
|  | Men | 718 | 37% | 47% | 35% | 37% | 40% | 27% | 0.010 |
|  |  |  |  |  |  |  |  |  |  |
| 11. Wait-time to see provider exceeded 45 minutes | All | 1297 | 14% | 13% | 14% | 15% | 17% | 9% | 0.235 |
|  | Women | 586 | 14% | 14% | 10% | 22% | 18% | 9% | 0.060 |
|  | Men | 711 | 12% | 10% | 18% | 7% | 17% | 9% | 0.024 |
|  |  |  |  |  |  |  |  |  |  |
| 12. Consultation time with provider was less than 15 minutes | All | 1293 | 63% | 58% | 58% | 69% | 70% | 64% | 0.007 |
|  | Women | 584 | 60% | 56% | 61% | 68% | 64% | 55% | 0.272 |
|  | Men | 709 | 66% | 62% | 56% | 72% | 75% | 74% | 0.001 |
|  |  |  |  |  |  |  |  |  |  |
| **Care experience over the past year** | | | | | | | | | |
| 1. Experienced medical error during treatment | All | 1403 | 5% | 8% | 5% | 3% | 3% | 2% | 0.003 |
|  | Women | 637 | 5% | 10% | 3% | 3% | 2% | 2% | 0.007 |
|  | Men | 766 | 5% | 5% | 8% | 3% | 3% | 2% | 0.077 |
|  |  |  |  |  |  |  |  |  |  |
| 2. Experienced discrimination or unfair treatment by a health worker | All | 1404 | 12% | 19% | 8% | 13% | 8% | 5% | <0.001 |
|  | Women | 637 | 15% | 24% | 7% | 17% | 13% | 8% | <0.001 |
|  | Men | 767 | 7% | 11% | 10% | 7% | 3% | 0% | 0.001 |

[1] ANOVA test for equality of proportions across age groups
