# Supplementary material for: Health system utilization and perceived quality among adults in Lao PDR: evidence from a nationally representative phone survey
Source: BMC Public Health. 2024 Feb 22;24:565. doi: 10.1186/s12889-024-18039-2 (PMC10882776; doi:10.1186/s12889-024-18039-2)
Supplement: Supplementary file 3 — Additional file 3. [file 12889_2024_18039_MOESM3_ESM.docx]

Additional file 3. Coverage of preventive health services by sex and age

| **Type of health examination** | **Sex** | **Estimated Lao population in age-sex group** | **N**  **(survey responses)** | **Total** | **By age** | | | | | **p-value [3]** |
| --- | --- | --- | --- | --- | --- | --- | --- | --- | --- | --- |
|  |  |  |  |  | 18-29 | 30-39 | 40-49 | 50-59 | 60+ |  |
| Mammogram [1] | Women | 576,589 | 268 | 12% | - | - | - | 15% | 10% | 0.1363 |
|  |  |  |  |  |  |  |  |  |  |  |
| Cervical cancer screening [2] | Women | 2,371,134 | 869 | 16% | 16% | 17% | 19% | 18% | 8% | 0.0973 |
|  |  |  |  |  |  |  |  |  |  |  |
| Vision check | All | 4,712,001 | 2007 | 18% | 14% | 19% | 19% | 23% | 18% | 0.0148 |
|  | Women | 2,371,134 | 870 | 18% | 11% | 15% | 20% | 32% | 25% | <0.001 |
|  | Men | 2,340,867 | 1137 | 18% | 18% | 24% | 19% | 16% | 11% | 0.0195 |
|  |  |  |  |  |  |  |  |  |  |  |
| Teeth check | All | 4,712,001 | 2007 | 16% | 13% | 17% | 17% | 20% | 15% | 0.079 |
|  | Women | 2,371,134 | 870 | 18% | 14% | 17% | 23% | 23% | 16% | 0.113 |
|  | Men | 2,340,867 | 1137 | 14% | 11% | 17% | 12% | 17% | 15% | 0.183 |
|  |  |  |  |  |  |  |  |  |  |  |
| Blood pressure | All | 4,712,001 | 2007 | 59% | 50% | 58% | 67% | 58% | 69% | <0.001 |
|  | Women | 2,371,134 | 870 | 62% | 55% | 58% | 73% | 68% | 66% | 0.002 |
|  | Men | 2,340,867 | 1137 | 55% | 44% | 59% | 61% | 49% | 72% | <0.001 |
|  |  |  |  |  |  |  |  |  |  |  |
| Blood glucose | All | 4,712,001 | 2006 | 35% | 22% | 34% | 43% | 44% | 50% | <0.001 |
|  | Women | 2,371,134 | 869 | 36% | 23% | 30% | 50% | 49% | 50% | <0.001 |
|  | Men | 2,340,867 | 1137 | 34% | 20% | 38% | 37% | 41% | 50% | <0.001 |
|  |  |  |  |  |  |  |  |  |  |  |
| Blood cholesterol | All | 4,712,001 | 2005 | 33% | 20% | 31% | 42% | 42% | 48% | <0.001 |
|  | Women | 2,371,134 | 868 | 36% | 21% | 30% | 47% | 49% | 54% | <0.001 |
|  | Men | 2,340,867 | 1137 | 31% | 20% | 33% | 37% | 36% | 41% | <0.001 |
|  |  |  |  |  |  |  |  |  |  |  |
| Mental health examination | All | 4,712,001 | 2007 | 1% | 0% | 0% | 2% | 0% | 1% | 0.0188 |
|  | Women | 2,371,134 | 870 | 1% | 0% | 1% | 2% | 0% | 0% | 0.0663 |
|  | Men | 2,340,867 | 1137 | 1% | 0% | 0% | 1% | 0% | 2% | 0.1884 |

^[[1]](#footnote-1)^[1] Women older than 50

^[[2]](#footnote-2)^[2] All women 18 and higher

[3] ANOVA test for equality of proportions across age group

1. [↑](#footnote-ref-1)
2. [↑](#footnote-ref-2)
