# Supplementary material for: Health system utilization and perceived quality among adults in Lao PDR: evidence from a nationally representative phone survey
Source: BMC Public Health. 2024 Feb 22;24:565. doi: 10.1186/s12889-024-18039-2 (PMC10882776; doi:10.1186/s12889-024-18039-2)
Supplement: Supplementary file 4 — Additional file 4. [file 12889_2024_18039_MOESM4_ESM.docx]

Additional file 4. Care experiences and unmet need for care by sex and ethnicity

|  | **Sex** | **N**  **(survey responses)** | **Total** | **Ethnic groups** | | | **p-value ^[1]^** |
| --- | --- | --- | --- | --- | --- | --- | --- |
|  |  |  |  | **Lao-Tai** | **Mon-Khmer** | **Hmong-mien** |  |
| 1. Experienced medical error during treatment | All ^[2]^ | 1375 | 5% | 5% | 6% | 5% | 0.839 |
|  | Women | 629 | 5% | 5% | 5% | 5% | 0.984 |
|  | Men | 746 | 5% | 4% | 6% | 5% | 0.684 |
|  |  |  |  |  |  |  |  |
| 2. Experienced discrimination or unfair treatment by a health worker | All ^[2]^ | 1376 | 12% | 11% | 12% | 14% | 0.584 |
|  | Women | 629 | 15% | 14% | 18% | 15% | 0.711 |
|  | Men | 747 | 7% | 6% | 8% | 13% | 0.117 |
|  |  |  |  |  |  |  |  |
| 3. Did not use care when needed (unmet need) | All ^[2]^ | 1958 | 16% | 16% | 21% | 12% | 0.014 |
|  | Women | 856 | 19% | 18% | 37% | 13% | <0.001 |
|  | Men | 1102 | 13% | 13% | 12% | 11% | 0.794 |

[1] ANOVA test for equality of proportions across ethnic groups

[2] Includes 3 main ethnicities only
